# Ammonia Oxidation Potentials and Ammonia Oxidizers of Lichen–Moss Vegetated Soils at Two Ice-free Areas in East Antarctica

**DOI:** 10.1264/jsme2.ME19126

**Published:** 2020-02-01

**Authors:** Kentaro Hayashi, Yukiko Tanabe, Nobuhide Fujitake, Morimaru Kida, Yong Wang, Masahito Hayatsu, Sakae Kudoh

**Affiliations:** 1 Institute for Agro-Environmental Sciences, National Agriculture and Food Research Organization, Tsukuba 305–8604, Japan; 2 National Institute of Polar Research, Tachikawa 190–8518, Japan; 3 SOKENDAI (The Graduate University for Advanced Studies), Tachikawa 190–8518, Japan; 4 Graduate School of Agricultural Science, Kobe University, Kobe 657–8501, Japan; 5 Research Fellow of Japan Society for the Promotion of Science

**Keywords:** ammonia-oxidizing archaea, ammonia-oxidizing bacteria, nitrification, substrate concentration response, temperature response

## Abstract

The maximum ammonia oxidation potential (AOP) of a topsoil in Langhovde, East Antarctica was 22.1±2.4‍ ‍ng N g^–1^ dry soil h^–1^ (2‍ ‍mM ammonium, 10°C, *n*=3). This topsoil exhibited twin AOP peaks (1 and 2‍ ‍mM ammonium) at 10°C, but not at 20°C. Six and ten operational taxonomic units (OTUs) were identified for ammonia-oxidizing bacteria (AOB) and archaea (AOA) *amoA*, respectively. AOB were classified into *Nitrosospira*; the two dominant OTUs corresponded to the Mount Everest cluster. AOA were classified into three clusters; *Nitrososphaera* and *Nitrosocosmicus* were the two dominant clusters.

Ice-free areas constitute 0.4% of the Antarctic continent (Cary *et al.*, 2010), which corresponds to a land area of *ca.* 56,000‍ ‍km^2^. Soils formed in these areas are permafrost-affected soils (Gelisols) (Bockheim and McLeod, 2006; 2008) and are characterized by microbially-driven nitrogen (N) cycling (Wynn-Williams, 1990; Cary *et al.*, 2010). Ammonia oxidation by bacteria and archaea is generally the rate-limiting step within the nitrification process in various soils (Prosser and Nicol, 2012). Research on the nitrification properties of Antarctic soils provides important information on N cycling in this extreme environment on Earth. However, the nitrification properties of Antarctic soils remain largely unknown (Jung *et al.*, 2011; Han *et al.*, 2013; Magalhaes *et al.*, 2014). Dronning Maud Land and Enderby Land in East Antarctica have not yet been studied, and there is currently no information on the vertical distribution of ammonia oxidation potential (AOP) in soils. We investigated the AOPs of East Antarctic soils, including the substrate dependency and genetic classification of ammonia-oxidizing bacteria (AOB) and archaea (AOA) in these soils.

Soil samples were collected at two plots (Y1 and Y2) in January 2017 in Yukidori-zawa, located in Langhovde, Dronning Maud Land (Y1, 69°14'28"S, 39°44'40"E; Y2, 69°14'26"S, 39°45'08"E), and at one plot (RL) in February 2017 near Mt. Riiser-Larsen, Enderby Land (66°46'18"S, 50°35'17"E). Each plot was covered with lichen and moss. AOP was defined as the nitrite production rate by an aerobic incubation with the addition of a substrate solution (Hayashi *et al.*, 2016). The substrate solution for a standard assay contained 1‍ ‍mM ammonium sulfate (corresponding to 2‍ ‍mM ammonium), 10‍ ‍mM sodium chlorate, and 1‍ ‍mM HEPES. Chlorate acts as an inhibitor of nitrite oxidation (Belser and Mays, 1980), whereas HEPES acts as a neutral pH buffer (Klotz, 2011). AOPs in fresh soil samples collected from Y1 and Y2 (*n*=3) during the field expedition were measured at approximately 10°C, whereas those for RL (*n*=3) were measured at 10 and 20°C in April 2017 in Japan. Further measurements of AOP using combinations of eight substrate concentrations (0.1, 0.2, 0.5, 1.0, 1.5, 2, 3, and 4‍ ‍mM ammonium) and two temperatures (10 and 20°C) were conducted on the topsoil of Y2 (depth of 0–3 cm), which exhibited the maximum AOP from the standard assay. ANOVA and Tukey’s multiple comparison tests (α=0.05) were performed for the measured AOPs using SAS Add-In for Microsoft Office (SAS Institute). We performed total DNA extraction, quantification, a pyrosequencing analysis, and sequence analysis with phylogenetic assignments of the ammonia monooxygenase subunit A (*amoA*) genes of AOB and AOA. Further methodological details are outlined in Supplementary Information.

In the standard assay conducted at approximately 10°C, the maximum AOP in each plot was in the topsoil (soil beneath the lichen–moss layer): 5.8±2.1 (mean±SD), 22.1±2.4, and 4.3±0.3‍ ‍ng N g^–1^ dry soil h^–1^ in the Y1, Y2, and RL plots, respectively (Fig. 1). These measured AOPs were similar to other values in the literature, including those reported in Antarctic bulk soils in West Antarctica (2.3‍ ‍ng N g^–1^ dry soil h^–1^, room temperature) (Jung *et al.*, 2011) and in High Arctic soils under *Salix polaris*–moss vegetation in Svalbard (1.1–14.1‍ ‍ng N g^–1^ dry soil h^–1^, 10°C) (Hayashi *et al.*, 2016). There were significant effects on AOP of the topsoil of Y2 by substrates and by the interaction between substrates and temperature (*P*<0.001) (Fig. 2). The twin AOP peaks at 1 and 2‍ ‍mM ammonium and 10°C were significantly higher than all other AOPs (*P*<0.001); however, these peaks were not present at 20°C. Meanwhile, AOP at 0.1‍ ‍mM ammonium and 20°C was significantly higher than that at 10°C (*P*<0.05). Soil pH was nearly neutral, ranging between 6.1 and 6.6 (Table 1). Soil total carbon (C) was low (<1%) in all soil layers, except in the topsoil of Y2 (1.9%). The topsoil C:N ratio ranged between 10.5 and 12.8, indicating active N turnover, such as N_2_O production, shown in High Arctic soils (Hayashi *et al.*, 2018). Ammonium as the essential substrate of nitrification was detected in all layers. The soil of RL exhibited a relatively high nitrate content, which accounted for 1% of total N. Approximately equal cell numbers of AOB and AOA were detected in the topsoil of Y2 (Fig. 3). AOB were more abundant than AOA in the three lichen–moss layers and the topsoil of Y1, but were less abundant in the topsoil of RL and subsoils. AOP and cell numbers were not significantly related to AOB or AOA. Ammonia oxidation rates under the given conditions may differ among species of ammonia oxidizers. In this case, cell numbers as the sum of plural species did not necessarily correlate with AOP.

Six operational taxonomic units (OTUs) were identified for AOB-*amoA*: all were classified into *Nitrosospira* (Fig. 4). OTU1 and OTU2 were dominant AOB (Fig. 5) and grouped into the Mount Everest cluster (Fig. 4) (Zhang *et al.*, 2009). AOB in the Mount Everest cluster were also reported from McMurdo Dry Valleys, Antarctica (Magalhaes *et al.*, 2014), Nelson Island, the Antarctic Peninsula (Han *et al.*, 2013), and Ny-Ålesund, Svalbard (Hayashi *et al.*, 2016). Ten OTUs were identified for AOA-*amoA* and classified into three clusters: *Nitrososphaera*, *Nitrosocosmicus*, and *cluster A* (Fig. 4). Three of these OTUs were grouped into the *Nitrososphaera* cluster; two of these, OTU1 and OTU4, were also reported from maritime Antarctica (Wang *et al.*, 2019). Six OTUs were grouped into the *Nitrosocosmicus* cluster, recently isolated from *Nitrososphaera* sister clusters (Lehtovirta-Morley *et al.*, 2016). OTU2 and AOA reported from Mount Everest (Zhang *et al.*, 2009) and the Antarctic Peninsula (Han *et al.*, 2013) were grouped into *cluster A*; this cluster has neither cultured representatives to date nor 16S rRNA gene taxonomic affiliations. The dominant OTUs of AOA varied by plot and layer (Fig. 5).

The topsoil of Y2 had the maximum AOP value (Fig. 1), the highest ammonium content among soil layers (Table 1), and similar cell numbers of AOB and AOA (Fig. 3) comprising two and four major OTUs, respectively (Fig. 5). The ammonia oxidizers in this topsoil appeared to have various favorable substrate concentrations and temperatures, as evidenced by the twin peaks in AOP at 1 and 2‍ ‍mM ammonium, present at 10°C, but not at 20°C (Fig. 2). Future challenges include elucidating the substrate and temperature dependencies of each of the AOB and AOA species, *in situ* annual changes in the nitrification process, and the contribution of each ammonia oxidizer to the *in situ* changes in nitrification under concomitant annual changes in temperature, water regime, and substrate conditions.

## Accession number

The sequences described in the present study have been deposited in the DNA Data Bank of Japan (accession number DRA008839).

## Supplementary Material

Supplementary Material

## Figures and Tables

**Fig. 1.**
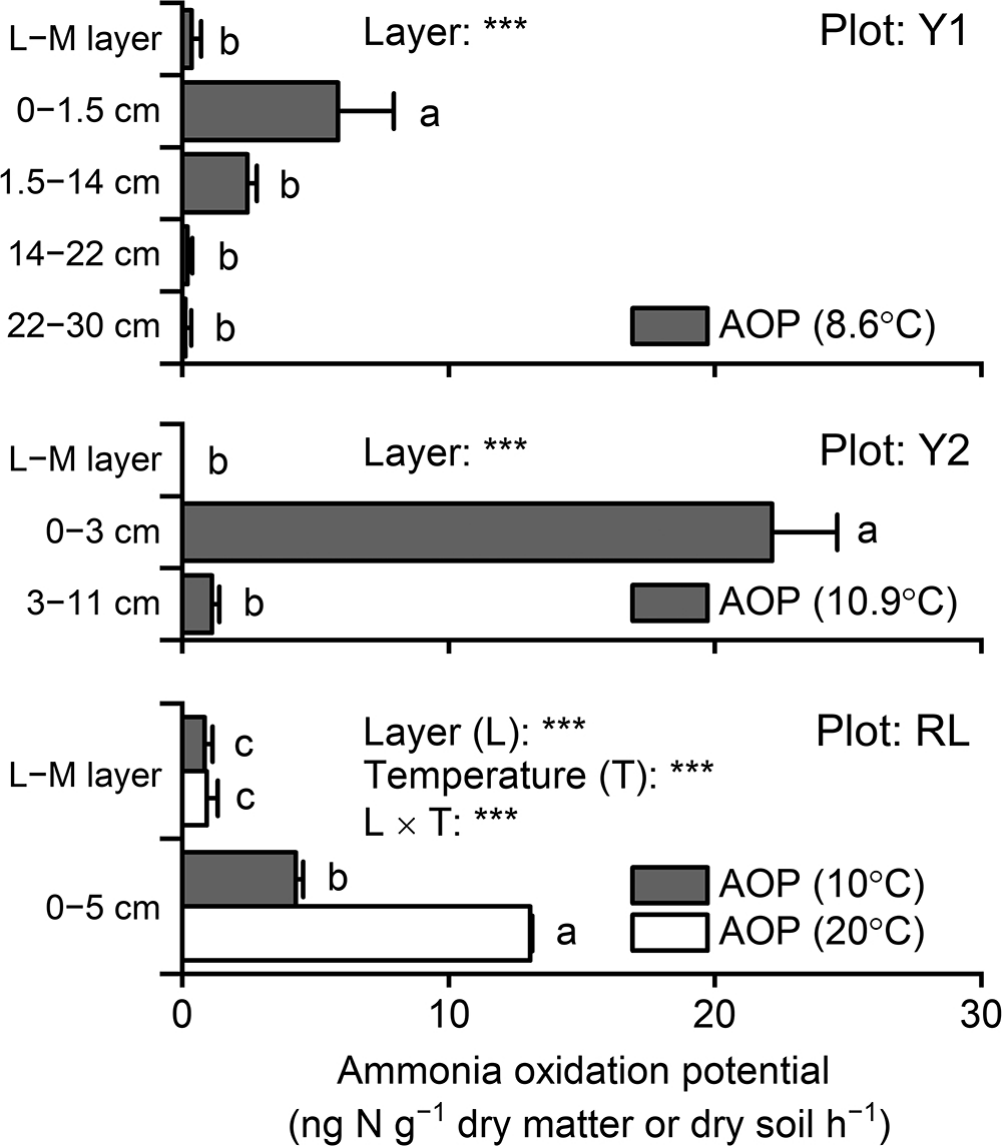
Ammonia oxidation potentials of lichen–moss and soil samples collected at three plots (Y1, Y2, and RL) assessed by a standard assay (2‍ ‍mM ammonium, *n*=3). Different letters indicate significant differences (*P*<0.05) between layers (Y1 and Y2) or layers and temperatures (RL). ***, significant effects or interactions (*P*<0.001).

**Fig. 2.**
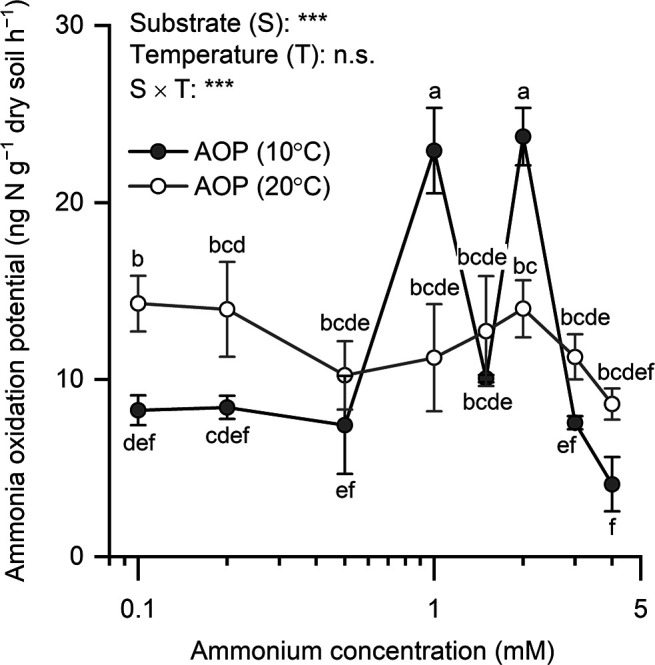
Ammonia oxidation potentials (AOPs) of the topsoil (depth of 0–3 cm) at the Y2 plot assayed for combinations of eight substrate concentrations and two temperature conditions. Symbols and whiskers denote mean values and standard deviations, respectively (*n*=3). Different letters denote significant differences (*P*<0.05) based on the significant interaction between substrates and temperature (*P*<0.001), followed by Tukey’s multi-comparison test. n.s., not significant.

**Fig. 3.**
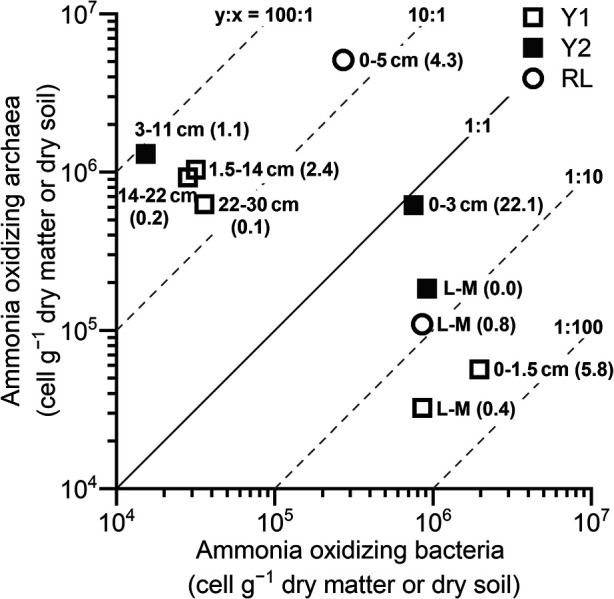
Cell numbers of ammonia-oxidizing bacteria (AOB) and archaea (AOA) *amoA* in each lichen–moss or soil layer assessed by quantitative PCR. L–M, lichen–moss layer on the topsoil. Values in parentheses denote AOP (ng N g^–1^ dry matter or dry soil h^–1^) as assessed by a standard assay (Fig. 1).

**Fig. 4.**
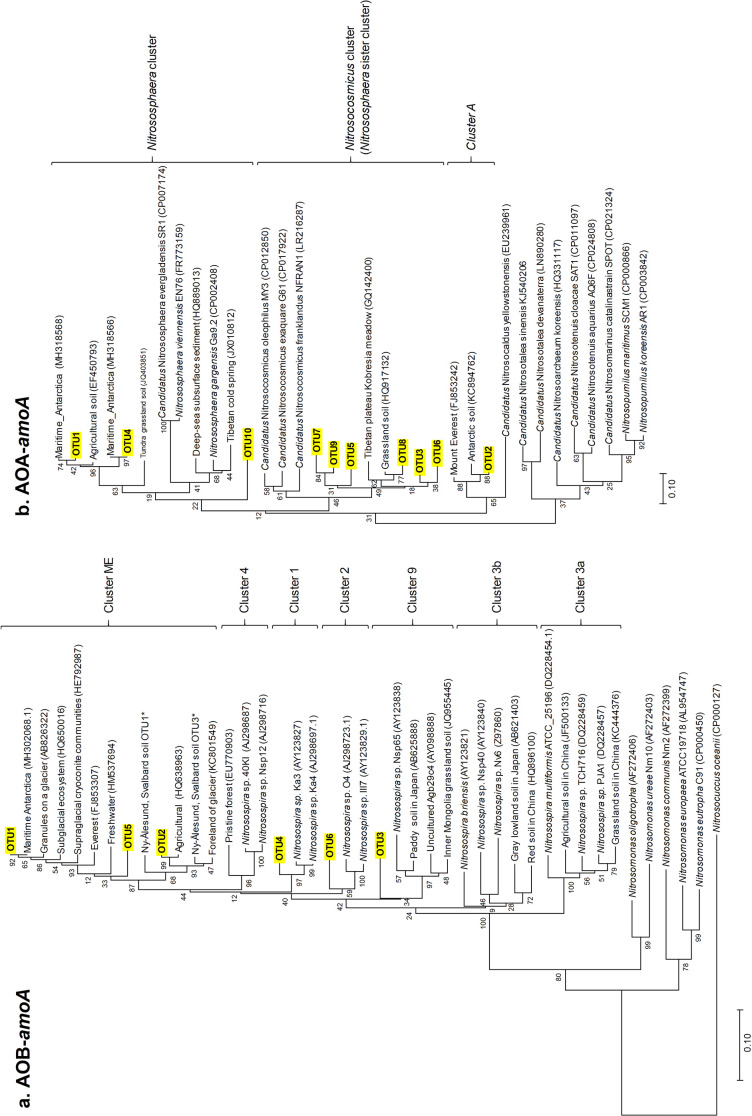
Phylogenetic trees of (a) ammonia-oxidizing bacteria (AOB) and (b) archaea (AOA) *amoA*. OTU, operational taxonomic unit. “Ny-Ålesund, Svalbard soil” OTUs were reported previously (Hayashi *et al.*, 2016). Cluster A corresponds to AOA with neither cultured representatives to date nor 16S rRNA gene taxonomic affiliations.

**Fig. 5.**
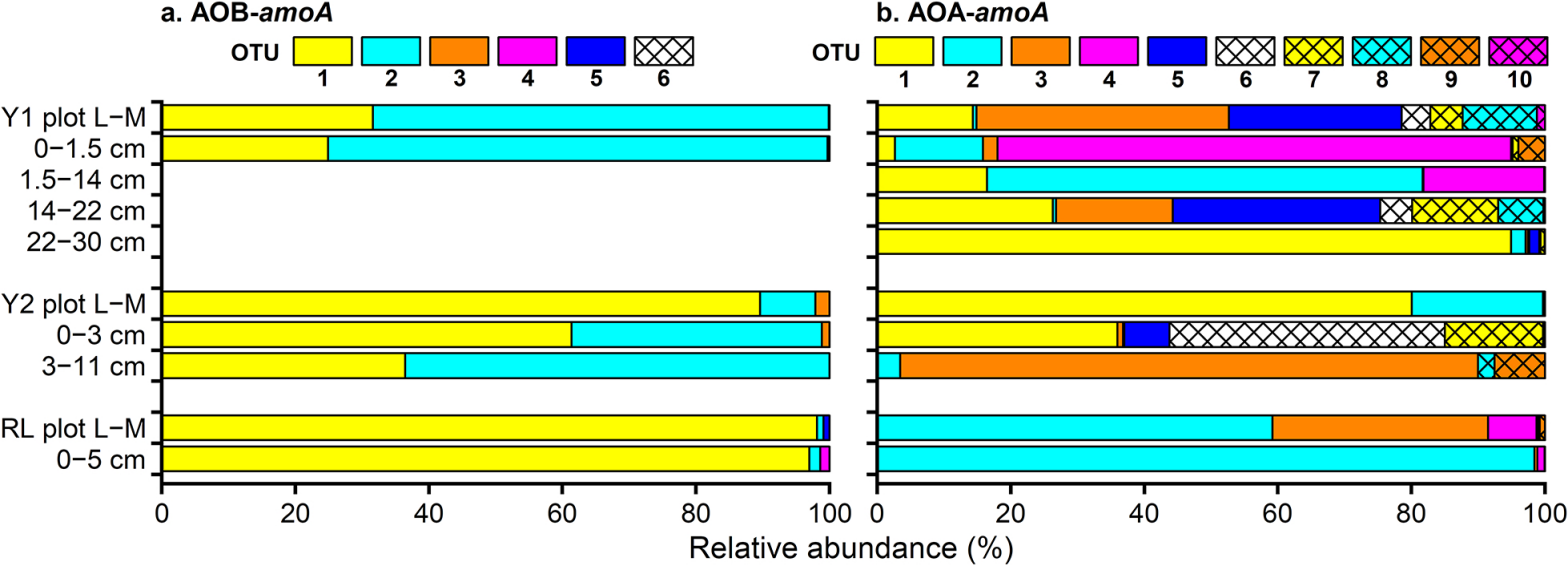
Relative abundance of (a) ammonia-oxidizing bacteria (AOB) *amoA* sequences and (b) archaea (AOA) *amoA* sequences. OTU, operational taxonomic unit; L–M, lichen–moss layer. Missing data on AOB (depths of 1.5–14 cm, 14–22 cm, and 22–30 cm at Y1) were due to insufficient amounts of purified DNA being collected.

**Table 1.** Soil properties. Values in parentheses denote standard deviations (*n*=3).

Plot	Layer	Soil pH (1:2.5 H_2_O)	Total carbon (%)	Total nitrogen (%)	Carbon-to-nitrogen ratio (w/w)	NH_4_-N (μg g^–1^)	NO_3_-N (μg g^–1^)
Y1	Lichen–moss	—	8.31 (0.02)	0.462 (0.011)	18.0 (0.4)	4.4 (0.1)	ND
	0–1.5 cm	6.1	0.70 (0.05)	0.057 (0.002)	12.2 (0.5)	2.0 (0.2)	0.99 (0.13)
	1.5–14 cm	6.6	0.12 (0.003)	0.012 (0.002)	10.0 (1.5)	1.5 (0.2)	0.20 (0.05)
	14–22 cm	6.5	0.064 (0.002)	0.004 (0.001)	18.8 (4.6)	1.6 (0.2)	0.15 (0.02)
	22–30 cm	6.4	0.18 (0.01)	0.014 (0.001)	12.4 (1.3)	1.4 (0.1)	0.19 (0.09)
Y2	Lichen–moss	—	16.6 (0.11)	0.701 (0.006)	23.7 (0.4)	6.7 (1.2)	ND
	0–3 cm	6.2	1.89 (0.02)	0.148 (0.003)	12.8 (0.2)	7.3 (0.5)	0.90 (0.01)
	3–11 cm	6.4	0.33 (0.05)	0.029 (0.006)	11.5 (0.9)	1.6 (0.1)	0.41 (0.09)
RL	Lichen–moss	—	13.0 (0.10)	0.651 (0.007)	19.9 (0.4)	10.1 (0.7)	ND
	0–5 cm	6.4	0.77 (0.02)	0.073 (0.001)	10.5 (0.3)	1.6 (0.1)	7.7 (0.03)

ND, not detected. Detection limits: 1‍ ‍μg C for total carbon, 1‍ ‍μg N for total nitrogen, 0.88‍ ‍μg N L^–1^ in solution for NH_4_-N, and 5.6‍ ‍μg N L^–1^ in solution for NO_3_-N.
