# Supplementary material for: Ammonia Oxidation Potentials and Ammonia Oxidizers of Lichen–Moss Vegetated Soils at Two Ice-free Areas in East Antarctica
Source: Microbes Environ. 2020 Feb 1;35(1):ME19126. doi: 10.1264/jsme2.ME19126 (PMC7104286; doi:10.1264/jsme2.ME19126)
Supplement: Supplementary file 1 — Supplementary Material [file 35_19126_s1.pdf]

Supplementary information

Ammonia oxidation potentials and ammonia oxidizer of lichen–moss vegetated soils at two ice-free areas in East Antarctica

Kentaro Hayashi, Yukiko Tanabe, Nobuhide Fujitake, Morimaru Kida, Yong Wang, Masahito Hayatsu, Sakae Kudoh

Research plots and soil sampling

Locations of the study sites are shown in Fig. S1. The mean temperature from January 2014 to December 2016 observed at a weather monitoring plot near Y1 and Y2 was –9.1 °C (Kudoh *et al.*, 2019). No meteorological information is available for RL. Soil properties in the Antarctic ice-free areas are affected by seabirds inhabiting the region (Ramsay and Stannard, 1986; Wynn-Williams, 1990; Cary et al., 2010). Yukidori-zawa is a valley where snow petrels (*Pagodroma nivea*) and south polar skuas (*Stercorarius maccormicki*) sparsely inhabit during the short summer period. Y1 and Y2 were both located on the lower part of a slope down to the valley; neither of these sites were near their nests. The nearest rookery of Adèlie penguins (*Pygoscelis adeliae*) to Y1 and Y2 located about 6 km northwest. RL is located on a gently-sloping rocky hill and was also not close to bird nests. The nearest rookery of Adèlie penguins with south polar skuas and a few emperor penguins

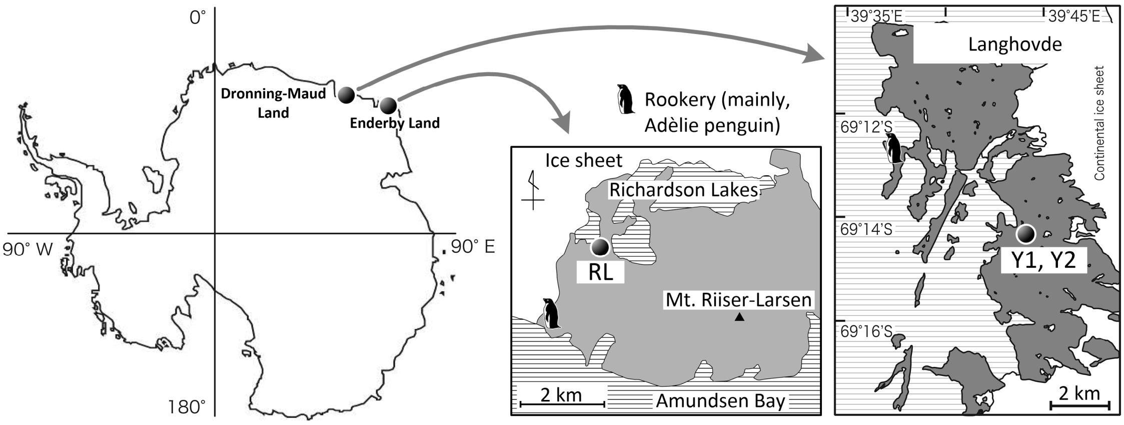

Fig. S1. Study sites in Antarctica where soil samples were collected.

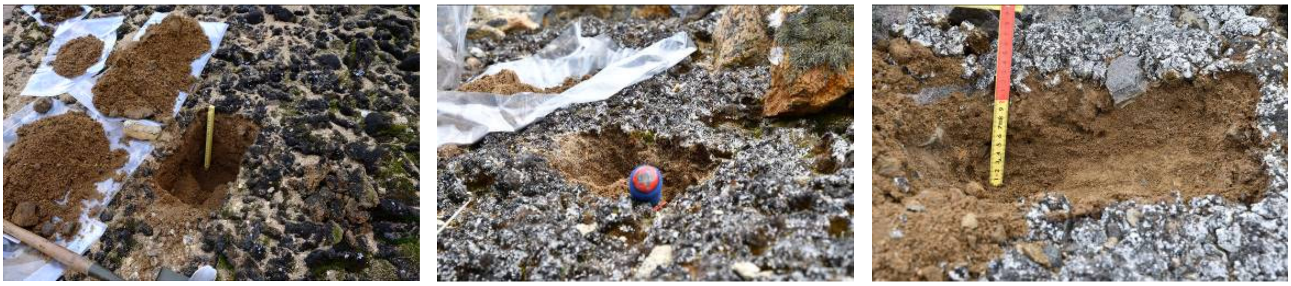

Fig. S2. Sampling plots at Y1 (left), Y2 (center), and RL (right).

(*Aptenodytes forsteri*) to RL located about 2.5 km southwest.

Soil sampling was conducted on 14 (Y1) and 15 (Y2) January and on 19 (RL) February 2017 as part of the 58th Japanese Antarctic Research Expedition. The lichen–moss layer and underlying soil layers were separately collected (Fig. S2). The soil at Y1 was sandy and over 30-cm deep. The soils at Y2 and RL were also sandy but thinly formed on rocks. The samples of Y1 plot consisted of the lichen–moss layer and soil layers of 0–1.5 1.5–14, 14–22, and 22–30 cm depths. Those of Y2 consisted of the lichen–moss layer and soil layers of 0–3 and 3–11 cm depths. Those of RL consisted of the lichen–moss layer and soil layer of 0–5 cm depth. Samples collected at Y1 and Y2 were divided into three portions, with each treated in one of the following ways: 1) kept fresh for immediate AOP measurements during the field expedition, 2) air-dried for later soil analysis, and 3) frozen for later microbial analysis in Japan. Fresh samples collected at RL during the backhaul of the icebreaker Shirase were similarly divided into two portions: 1) kept fresh for later AOP analysis in Japan, and 2) frozen for later soil and microbial analyses in Japan. The frozen RL samples for soil analysis were air-dried in Japan that started on 18 April 2017.

For AOP measurements, the Y1 samples were analyzed within the day of sampling. The Y2 samples were stored at the outdoor temperatures in the field for 7 days until AOP analysis. The RL samples were stored at cold conditions (ca. 6 °C) for 63 days until AOP analysis, for which a chill room in the icebreaker Shirase, a cold box to transport the sample to the laboratory in Japan, and a chill room in the laboratory were used. The Y1 and Y2 samples for soil analysis were stored at the outdoor temperatures in the field for 2 and 1 days, respectively, until the beginning of air-drying. The RL samples for soil analysis were frozen for 58 days until the beginning of air-drying.

AOP measurements

A temperature of 20 °C was selected as an incubation temperature, because many literature data of soil AOP in other regions of the world were obtained at 20 °C (e.g., Hayashi *et al.*, 2016 for High Arctic). However, a temperature of 20 °C seemed too high that the current Antarctic soils have never experienced, whereas summer air temperatures in the working spaces in the field were frequently higher than 5 °C. Therefore, 10 °C was also selected as an incubation temperature.

The samples of Y1 and Y2 underwent aerobic static incubation on a smaller scale than usually conducted given the limited analytical resources available during the field expedition. The AOP measurement of Y1 samples was conducted at Yukidori Hut, a remote base located in Langhovde, Dronning Maud Land, ca. 27 km distant from Syowa Station. That of Y2 samples was conducted at another base, Kizahashi Hut, located in Skarvsnes, Dronning Maud Land, ca. 52 km distant from Syowa Station. Fresh sample (0.5 g, 2-mm sieved) was added to a 2-mL centrifugation tube, and 1 mL of the substrate solution was added. The tube was shaken with a tube shaker to create a suspension, 200 μL of which was immediately added to a 500-μL centrifuge tube. The remaining suspension in the 2-mL tube was put in a cold box for static incubation at temperatures around

10 °C. The 500-μL tube was centrifuged at 12,000 rpm for 10 min, and 50 μL of the supernatant was added to a sample well of a microplate (96 wells). The nitrite concentration in the supernatant at zero time was immediately determined with a colorimetric method using N-(1-naphthyl) ethylenediamine. A portable microplate reader (iMark, Bio-Rad) was used to determine the nitrite concentration. The Y1 and Y2 samples were statically incubated for 21 h (14–15 January 2017, 8.6 °C on average) and 17 h (22–23 January 2017, 10.9 °C on average), respectively, after which a second supernatant was prepared to determine the final nitrite concentration with the same methods outlined above. The nitrite production rate per unit weight of dry matter for the lichen–moss layer (ng N g^{-1} dry matter h^{-1}) or soil (ng N g^{-1} dry soil h^{-1}) was then defined and calculated as the AOP with the measured soil water content of the fresh soil (n = 3). Substrate and temperature dependency of AOP for the topsoil of Y2 (0–3 cm depth), *i.e.*, eight substrate concentrations (0.1, 0.2, 0.5, 1.0, 1.5, 2, 3, and 4 mM ammonium) and two temperatures (10 and 20 °C), was measured with 10 h incubation on 24 January 2017 (n = 3). Two thermostatic tube mixers were used to enable incubation at constant temperatures, for which electricity was supplied using a power generator during the field expedition.

A similar procedure was applied for the RL samples, except that 2.5 g of the fresh sample and 10 mL of the substrate solution were added to a 50-mL tube, and 1 mL of suspension to the centrifuge. The static incubation for the RL samples was conducted at a constant temperature of 10 or 20 °C for 20 h on 23–24 April 2017 in our laboratory in Japan.

We confirmed effects of incubation scales on obtained AOP values using a soil collected at an upland field in the Institute for Agro-Environmental Sciences, NARO, Japan, which exhibited the same order of AOPs compared to those of the Antarctic soils in the present study. As a result, no significant difference in AOP was found between the two scales, i.e., a standard scale and its one-tenth scale, according to t-test (n = 5, P = 0.376). It is, however, noted that more careful analytical operations are required for such the small-scale incubation.

Soil analysis

Air-dried samples were passed through a 2-mm sieve and then analyzed to determine soil pH, total carbon (C), total nitrogen (N), ammonium, and nitrate content per unit weight of dry matter (lichen–moss samples) or dry soil (soil samples) (n = 3). Carbon-to-nitrogen (C:N) ratios on a mass basis were also calculated. Water content of the air-dried samples used to calculate soil properties per unit weight of dry matter or dry soil was determined by weighing before and after drying in an oven at 105 °C for 12 h.

Soil pH was measured for a 1:2.5 solution of soil to water by weight.

Total C and N content was measured for both the lichen–moss and soil samples with an elemental analyzer (PE2400 Series II CHNS/O Analyzer, PerkinElmer). Precisely weighed (40–60 mg) air-dried samples were respectively packed into a tin capsule (8×5 mm, Exeter Analytical, Inc.).

Analytical conditions were as follows: standard substance, purified acetanilide; combustion temperature, 950 °C; and reduction temperature, 640 °C.

Ammonium and nitrate content was measured for both the lichen–moss and soil samples. For ammonium content, 3 g of the air-dried sample was extracted with 30 mL of 10% (w/w) potassium chloride solution by shaking for 30 min. After several minutes of still standing, the supernatant was passed through a qualitative filter (No. 2, ADVANTEC), and then the ammonium concentration was determined by an indophenol method. For nitrate content, 1 g of the air-dried sample was extracted with 10 mL of pure water by shaking for 1 h and then centrifuged at 9000 rpm for 5 min. The supernatant was passed through a membrane filter with a pore size of 0.45 μm (13HP045CN, ADVANTEC), and then the nitrate concentration was determined with an ion chromatograph (IC-2001, Tosoh).

DNA extraction from soil

Total soil DNA was extracted and purified with a FastDNA spin kit for soil (Qbiogene/MP Biomedicals, Solon, OH, USA) without skim milk and with a DNA Clean and ConcentratorTM-25 kit (Zymo Research Corp., Orange, CA, USA).

Quantification of AOB- and AOA-*amoA* gene

AOB and AOA-*amoA* gene copy numbers were quantified by using a SYBR Green I-based real-time PCR technique. The primer sets *amoA*-1F (5′-GGG GTT TCT ACT GGT GGT-3′) (Rotthauwe *et al.*, 1997) and *amoA*-2IR (5′-CCC CTC IGI AAA GCC TTC TTC-3′) (Avrahami *et al.*, 2003) were used for AOB-*amoA*; AOA *amoA*19IF (5′-ATG GTC TGG CTI AGA CG-3′) (Morimoto *et al.*, 2011) and CrenamoA616r (5′-GCC ATC CAT CTG TAT GTC CA-3′) (Tourna *et al.*, 2008) were used for AOA-*amoA*. Real-time PCR was performed on a StepOne Plus real-time PCR system (Applied Biosystems, Foster City, CA, USA). Quantitative PCR for AOB-*amoA* and AOA-*amoA* was performed under the same conditions outlined in Hayashi *et al.* (2016).

High throughput sequencing

AOB and AOA-*amoA* genes were amplified by 25 PCR cycles using the respective primers of *amoA*-1F and *amoA*-2R-GG (Nicolaisen and Ramsing, 2002), and GenAOAF and GenAOAR (Meinhardt *et al.*, 2015); an adaptor sequence was used for both genes. Once the PCR products were purified with AMPure beads, an 8-cycle PCR was performed with primers containing a unique 8 bp-index sequence to mark samples. The purified products were pooled in equimolar and paired-end sequences (2 × 300) on an Illumina MiSeq platform (Illumina, SanDiego, CA). This *amoA* sequencing was outsourced to Seibutsu Giken Co., Ltd (Kanagawa, Japan).

Sequence analysis bioinformatic pipeline

The FASTQ files corresponding to AOB and AOA-*amoA* were processed with an R package DADA2 (version 1.10.1) (Callahan *et al.*, 2016) and Mothur (version 1.42.2) (Schloss *et al.*, 2009). All FASTQ files were quality inspected with DADA2. In order to remove the low-quality 3'-end, all sequences were trimmed to 120 nt in both forward and reverse sequence files for AOA-*amoA*, and 295 nt in forward and 225 nt in reverse sequence files for AOB-*amoA*. Then, sequences were denoised, followed by dereplication of sequences and removal of chimeras by using DADA2. The sequences generated from DADA2 were clustered into operational taxonomic units (OTUs) with a cut-off value of 0.07 for AOA-*amoA* and 0.05 for AOB-*amoA* by using Mothur as previously described (Tago *et al.*, 2015). Local BlastN search against reference *amoA* sequences, which were collected from the NCBI database, was performed to decide sequence identity by using NCBI Blast+. Illumina MiSeq sequence data associated with this study has been deposited in DDBJ under the primary accession number DRA008839.

Phylogenetic assignment

The obtained AOB and AOA-*amoA* sequences were analyzed with MEGA7 software (Tamura *et al.*, 2013). Reference sequences of *amoA* genes were obtained from NCBI. The sequences were aligned by using the ClustalW program (Chenna *et al.*, 2003). Each of the AOB and AOA-*amoA* phylogenic trees was constructed by using the neighbor-joining method and assessed with 100 bootstrap replicates.

Note to the phylogenetic features

For AOB-*amoA*, six OTUs were identified in the present study (Fig. 4), all of which were classified into *Nitrosospira*. Other major AOB (*i.e.*, *Nitrosomonas* and *Nitrosococcus*) were not identified. OTU1, OTU2, and OTU5 were grouped into the Mount Everest cluster (Zhang *et al.*, 2009).

For AOA-*amoA*, ten OTUs were identified in the present study (Fig. 4). Pester *et al.* (2012) suggested four clusters (*i.e.*, *Nitrosocaldus*, *Nitrosopumilus*, *Nitrososphaera*, and *Nitrosotalea* clusters) and *Nitrososphaere* sister clusters for AOA. Recently, the *Nitrosocosmicus* cluster was isolated from the *Nitrososphaere* sister clusters (Lehtovirta-Morley *et al.*, 2016). *Nitrososphaera* clusters are detected in various environments including soils, and sequences within *Nitrososphaere* sister clusters are widely distributed in soil and other environments (Gubry-Rangin *et al.*, 2011; Pester *et al.*, 2012). In the present study, OTUs classified into the *Nitrososphaere* cluster, *Nitrosocosmicus* cluster, and *cluster A* were identified. *Cluster A* corresponds to AOA with neither cultured representatives to date nor 16S rRNA gene taxonomic affiliation.

References

Avrahami, S., Liesack, W., and Conrad, R. (2003) Effects of temperature and fertilizer on activity and community structure of soil ammonia oxidizers. *Environ Microbiol* **5**: 691–705.

Callahan, B.J., McMurdie, P.J., Rosen, M.J., Han, A.W., Johnson, A.J., and Holmes, S.P. (2016) DADA2: High-resolution sample inference from Illumina amplicon data. *Nat Methods* **13**: 581–583.

Cary, S.C., McDonald, I.R., Barrett, J.E., and Cowan, D.A. (2010) On the rocks: the microbiology of Antarctic Dry Valley soils. *Nat Rev Microbiol* **8**: 129–138.

Chenna, R., Sugawara, H., Koike, T., Lopez, R., Gibson, T.J., Higgins, D.G., and Thompson, J.D. (2003) Multiple sequence alignment with the Clustal series of programs. *Nucleic Acids Res* **31**: 3497–3500.

Gubry-Rangin, C., Hai, B., Quince, C., Engel, M., Thomson, B.C., James, P., Schloter, M., Griffiths, R.I., Prosser, J.I., and Nicol, G.W. (2011) Niche specialization of terrestrial archaeal ammonia oxidizers. *Proc Natl Acad Sci USA* **108**: 21206–21211.

Hayashi, K., Shimomura, Y., Morimoto, S., Uchida, M., Nakatsubo, T., and Hayatsu, M. (2016) Characteristics of ammonia oxidation potentials and ammonia oxidizers in mineral soil under *Salix polaris*–moss vegetation in Ny-Ålesund, Svalbard. *Polar Biol* **39**: 725–741.

Kudoh, S., Tanabe, Y., Hayashi, K., Kida, M., Fujitake, N., Uchida, M., and Imura, S. (2019) Meteorological data from ice-free areas in Yukidori Zawa, Langhovde and Kizahashi Hama, Skarvsnes on Sôya Coast, East Antarctica during December 2014–December 2016. *Polar Data J* **3**: 37–45.

Lehtovirta-Morley, L.E., Ross, J., Hink, L., Weber, E.B., Gubry-Rangin, C., Thion, C., Prosser, J.I., and Nicol, G.W. (2016) Isolation of '*Candidatus* Nitrosocosmicus franklandus', a novel ureolytic soil archaeal ammonia oxidiser with tolerance to high ammonia concentration. *FEMS Microbiol Ecol* **92**: fiw057.

Meinhardt, K.A., Bertagnolli, A., Pannu, M.W., Strand, S.E., Brown, S.L., and Stahl, D.A. (2015) Evaluation of revised polymerase chain reaction primers for more inclusive quantification of ammonia-oxidizing archaea and bacteria. *Environ Microbiol Rep* **7**: 354–363.

Morimoto, S., Hayatsu, M., Hoshino, Y.T., Nagaoka, K., Yamazaki, M., Karasawa, T., Takenaka, M., and Akiyama, H. (2011) Quantitative analyses of ammonia-oxidizing archaea (AOA) and ammonia-oxidizing bacteria (AOB) in fields with different soil types. *Microbes Environ* **26**: 248–253.

Nicolaisen, M.H., and Ramsing, N.B. (2002) Denaturing gradient gel electrophoresis (DGGE) approaches to study the diversity of ammonia-oxidizing bacteria. *J Microbiol Methods* **50**: 189–203.

Pester, M., Rattei, T., Flechl, S., Gröngröft, A., Richter, A., Overmann, J., Reinhold-Hurek, B., Loy, A., and Wagner, M. (2012) *amoA*-based consensus phylogeny of ammonia-oxidizing

archaea and deep sequencing of *amoA* genes from soils of four different geographic regions. *Environ Microbiol* **14**: 525–539.

Ramsay, A.J., and Stannard, R.E. (1986) Numbers and viability of bacteria in ornithogenic soils of Antarctica. *Polar Biol* **5**: 195–198.

Rotthauwe, J.H., Witzel, K.P., and Liesack, W. (1997) The ammonia monooxygenase structural gene *amoA* as a functional marker: molecular fine-scale analysis of natural ammonia-oxidizing populations. *Appl Environ Microbiol* **63**: 4704–4712.

Schloss, P.D., Westcott, S.L., Ryabin, T., *et al.* (2009) Introducing mothur: open-source, platform-independent, community-supported software for describing and comparing microbial communities. *Appl Environ Microbiol* **75**: 7537–7541.

Tago, K., Okubo, T., Shimomura, Y., Kikuchi, Y., Hori, T., Nagayama, A., and Hayatsu, M. (2015) Environmental factors shaping the community structure of ammonia-oxidizing bacteria and archaea in sugarcane field soil. *Microbes Environ* **30**: 21–28.

Tamura, K., Stecher, G., Peterson, D., Filipski, A., and Kumar, S. (2013) MEGA6: Molecular Evolutionary Genetics Analysis version 6.0. *Mol Biol Evol* **30**: 2725–2729.

Tourna, M., Freitag, T.E., Nicol, G.W., and Prosser, J.I. (2008) Growth, activity and temperature responses of ammonia-oxidizing archaea and bacteria in soil microcosms. *Environ Microbiol* **10**: 1357–1364.

Wynn-Williams, D.D. (1990) Ecological Aspects of Antarctic Microbiology. *Adv Microb Ecol* **11**: 71–146.

Zhang, L.M., Wang, M., Prosser, J.I., Zheng, Y.M., and He, J.Z. (2009) Altitude ammonia-oxidizing bacteria and archaea in soils of Mount Everest. *FEMS Microbiol Ecol* **70**: 208–217.
